# Physiology of Preaching

**Published:** 1869-05

**Authors:** 


### HALL'S
### JOURNAL OF HEALTH.

VOL. XVI. MAY, 1869. NO. 5.

### PHYSIOLOGY OF PREACHING.

HIGH bodily health is an important element of mental vigor, of clear thought, of logical accuracy, and of individual personal effort, influence and efficiency. A sickly man makes sickly arguments and is an unsafe guide. Imperfect health indicates imperfect blood, and as the brain feeds upon the blood, is nourished by it, its operations, under the influence of this depraved nourishment, must be prejudicially affected. He who drinks beer, thinks beer. When Byron wrote, he drank incredible quantities of brandy; less than every half hour through the live-long night he is said to have swallowed the fiery fuel, and what writer of his time has done more to corrupt the moral sense of his readers? profane, indecent, and disgusting, by turns. Burns was a drunkard, and drunkards and fast men, to this day, celebrate his birth with songs and wine. He was a maudlin sentimentalist; brilliant, indeed, but a great deal of his brilliancy was at the expense of good men, of the Church, of society and of religion, and better had it been for the world had Byron and Burns never lived.

To preach well, a man should be well. It is more necessary than in any other calling, that a clergyman should have vigorous, robust health, because his themes are immortal. Upon the power of his arguments, the convincingness of his logic and the clear statement of his positions, eternal destinies depend. Not that weakly, sickly men have not at times accomplished great things; but what greater and more perfect might they not have achieved, had they not been compelled to breast the tide of bodily infirmity.

### BEECHER—HALL—SPURGEON.

These are now the magnates of the Protestant pulpit. We have heard them all within a year; no men living so continuously draw large and admiring crowds. They are all men of "strong mind," of remarkable physical vigor and health; they may average two hundred pounds avoirdupoise. Two things may be safely said of them: they can appreciate a good dinner, but never have had a "feeling sense" of the horrors of dyspepsia; enjoying high health, they are enabled to accomplish an amount of clerical labor which to many would appear incredible if it were stated.

### SOURCE OF RENOWN.

The pre-eminence of these men does not depend so much on the greatness of their intellect, as upon the manner of their using it. Healthful in body, their brain works healthfully, strongly, clearly, legitimately; not brilliantly, but plainly and convincingly. Very few men are as far from even attempting an oratorical flourish, and yet listening multitudes hang upon their utterance with a deep delight. What millions of tears have been shed as these men have been speaking; though often, in the preaching of Hall, there is something too deep for tears, and the impression lasts longer—it follows for months. All of them are easily understood—you hear them easily. The powers of the attention are not wasted in catching the words; in threading the long sentences; in connecting the links of the argument: these all follow of themselves because their brains work clearly and well.

### THE VOICE.

We once asked the Rev. Dr. James W. Alexander, just returned from abroad, what he thought was the great secret of Spurgeon's fame. "He is a good man and has a good voice." Neither of the men named have a loud utterance, but they are strikingly alike in the distinctness of their enunciation. Neither of them are ever at a loss for words; they do not speak rapidly, nor very slow, but every syllable is distinctly and clearly pronounced. The want of this is a lamentable defect in three clergymen out of four in the United States. If not very near, you have to guess at a great part of what they say; high, low, seems to be the general defect. The first words of a sentence, and often the first syllables of a word, are "sky-high" with an almost whispering close, or such a running together of sounds as if determined to make their enunciation as incomprehensible as possible, and intelligent hearing is a pain. Hall has great freedom of utterance—not very rapid either, but every word is so completely and distinctly enunciated, and seems to follow so naturally and so appropriately, that it is a positive delight to hear him speak on any subject. Spurgeon's tabernacle can hold seven thousand men; it seats five thousand and yet every word is distinctly heard by the vast assembly. There is a clear musical ring in Beecher's voice, but between each word their is an appreciable interval of silence. Many of our clergymen are mere mumblers,—as if they had not physical strength to complete a sentence. It is not a loud voice that makes hearing easy; begin on a lower key, end on a higher one, and the average of strength expended will not be greater, while the pleasure of hearing will be greatly increased, and there is absolute rest between the words; that rest, short as it is, gives power to the next word. The busy heart beats always, beats ever, from the first instant of existence, until it ceases forever in the grave, yet between the beats there is such an appreciable quietude that physiologists have computed that for one-third of the time it is in actual rest, otherwise it would cease its action in a day. This is a law pertaining to all muscular motion; hence, he who will enunciate each syllable and word with a sharp and clear distinctness can speak longer with less fatigue. It is the plainest dictate of common sense that if you want to persuade a man to do anything for you, he must hear what you want, and hear it certainly, clearly, and without an effort.

HAVE A CLEAR IDEA

Of what you want to say to an assembly, if you wish to secure a successful hearing: the whole thought should be as clear, complete and defined in the " mind's eye " as the utterance of it should be distinct. Neither of the men named ever seem by any possibility to be in want of words; let a public speaker get an idea and feel its importance and no words need to be studied. The habiliments of the thoughts, fitting and apt, will spring spontaneously to the occasion. But a " muddy " brain can never have any other than ideas " clear as mud." The blood must be sparkling and pure and fresh as it leaps from heart and lung to the ever busy brain; but it is only pure blood that can flow healthfully. It may be instructive to glance at the points of agreement between the eminent men of whom we have been speaking, and the young men who read this, and propose entering professional life whether as clergymen, lawyers or politicians, might do well to take note of them.

They have high physical health.

They have strong, well-knit frames.

They have great distinctness of enunciation.

They are earnest men.

They are classically educated.

Here are five acquirable requisites, and those who do not have them all in liberal measure would do well to consider maturely whether they are not more loudly called to an humbler sphere than the professions. Be a " king in your calling if it is only to be a boot-black."

A GREAT PREACHER

Is the want of many communities; every one-horse town in the nation thinks itself an "important point;" that there are circumstances connected with it which imperatively require that it should have a man of superior talents, to " go in and out before them." The spread of the Christian religion is a work of human instrumentality under the guidance of the great Head of the Church, and the cause of religion is to be promoted like any other cause, as far as man is concerned, and with limitation it is a matter of dollars and cents expended judiciously, and with a pure motive; and the older the world gets, the more imperative does the need of money become; we are speaking now to the educated men of all sects; to scoffers and infidels and fast men and women who are without God and without hope in the world, we have nothing to say just now, except that God give you a better mind before you are called hence. It is by the "foolishness of preaching" that men and women are made to believe, and "and how can they hear without a preacher, and how shall they preach except they be sent?" In no country can all go to the wars; those only who are fit to fight must be sent, and the remainder must stay behind and furnish and feed those who go to the front. But those who do go to the front, to stand in the gap of death are no more bound to give their time to the country than those who remain; if they do give that time, those at home are as patriots bound to pay them for it. So in the Christian warfare; the men who are sent to the front, who are expected to do the hard work, almost all the work, the clergy, should be amply, liberally paid; so largely so, that they need not give one moment's thought or attention to any other than a clergyman's duty. They are the leaders, the captains of the hosts of Israel. What would have been thought of it if Washington had been so poorly paid as to be compelled to buy his food and clothing on credit, to give his note for it, and then depend on his relations to furnish the money to redeem it, and in default let it go unpaid. At this very moment half the clergy of the United States have to resort to their own private means to eke out a subsistance to make both ends meet, whereas if they had embarked in business their natural talents and their mental culture would have placed them in positions which would command fifty times the salaries now paid them. The clergy of this country, men of culture and classical education, do not average a salary of six hundred dollars a year—a sum less than a New York drayman, hod-carrier or omnibus driver *demands*, yes, DEMANDS; while the mass of the clergy receive their pay from hands which give it as a gratuity. Nine-tenths of those who do not pay the preacher in the roundabout way of pew-rents feel that it is so much given as charity. It is an outrage against all intelligence. A merchant gave his porter eight hundred dollars last year and on getting him a wife he said to him recently, "Patrick, you can 't support a wife and child on your salary, you must have a thousand after this." There are many book-keepers in New York who work from ten to four, and get two thousand dollars a year. The presidents of Insurance Companies, of even moderate standing, get five thousand dollars a year. Some get fifteen thousand, and give about two hours a day to their duties. The manager of a sugar refinery gets twenty thousand dollars a year. The Erie Railroad has paid its president twenty-five thousand dollars a year, and yet if a clergyman of greater talents, of a tenfold higher culture and moral character, is paid four or five thousand a year, it is counted a large salary. We admire the manliness and moral courage of a Presbyterian clergyman who, when the wants of the Educational Society were laid before the Presbytery, rose in his place and said, last week, "I cannot aid this society myself to the extent of a single dollar, nor do I feel called upon to present the object to my people. I demand of the Church that it shall liberally support the clergymen it already has. I have two sons, young men of promise and piety, but I cannot conscientiously advise them to study for the ministry; if the Church cannot command talent by generous salaries, talent will go where these can be commanded; with what face can I encourage a young man to study for the ministry, when I know that as a minister he could n't command the wages of a stage driver or a hotel waiter."

Hall's church in Fifth Avenue gave forty thousand dollars last year to make more young preachers; it gave twenty thousand dollars in one day to build more churches or some kindred object. Why, we have more churches now than we can fill and more preachers than we support. There are thousands of places in the United States where there are too many churches by threefold and even more. There is many a village which has four or five churches where there ought to be but one congregation ; one good sized house would hold comfortably all who attend the five, a clear loss to the Church of four clergymen ; there is no such waste of power in the management of our banks, our railroads, our insurance companies, and our mammoth business houses. There ought to be some remedy for these wastes studied out, in the light of the fact that many a widow's mite is contributed to pay these five men for doing the work which one could do better and more easily, which dollar was the result of weeks and months, it may be, of penny savings. We freely pronounce it a shame, an outrage and a dishonesty to have two churches in one village where there is not pecuniary ability to sustain one decently, respectably, generously ; starving, and thus degrading two ministers ; an old and a new school Presbyterian ; a high and low Episcopalian ; a northern and southern Methodist ; an open and close communion Baptist : fie upon ye, men of Israel ! "To what purpose is this waste ?" when there are so many fields white unto the harvest without one solitary laborer to put in his sickle to save the perishing grain.

### MAGNETIC INFLUENCE.

One man will rise to address an assembly and in an instant, before he utters a word, there will be a prepossession in his favor which takes possession of every hearer; another will strike a chill through every heart, and hence his efforts will be expended before the people begin to warm up to him. Beecher, before he utters a word, throws out the magnetism of a geniality which inspires a kind of cheeriness, and every man woman and child is wide awake for the first word, which by the genius of the man is sure to be such that each successive one prepares for the more greedy reception of the next following. There is a tenderness and a loving spiritual concern in the expression of Hall's face which wins the heart on the instant. Spurgeon has neither of these, but as he speaks, you draw closer and closer to him, and before you know it, you, with six or seven thousand others, are drawing a long breath, the intensity of your attention on his utterance having been such that you had seemingly ceased to breathe for a long time. This magnetic influence possessed in such lavishness by these men, we believe, is an acquisition, and has three elements—

1. Their intellects are persuaded of the truth of their mission.

2. Their steady contemplation of it, and their responsibilities have made eternal themes ever-present and felt realities.

3. Their hearts are baptised with love to God and man.

Thus, to be a power in the church, to win souls, in increasing crowds, it is necessary to see, to feel, to act in consistency with one another; it is these which make the preaching of the gospel the wisdom of God and the power of God. To preach successfully, then, as far as human agencies are concerned, a man must have good health, and a good heart; and that men ought not to excuse themselves from entering the ministry because they have not extraordinary mental endowments, we have only to remember that the conversion of souls is not by intellectual "might nor power, but by my Spirit, saith the Lord." That Spirit following the earnest enunciation of truths made clear as noon-day by the active workings of a brain, fed by blood, healthy, sparkling, life-giving, made thus healthy by a temperate, busy life, and by a mind kept at perfect ease by the certainty of means abundant enough for all domestic purposes, and for the acquisition of such aids to study as are found in the newest books and and in the best conducted magazines and newspapers, for to expect a minister to preach with power without affording him the means of using the current literature of the day is an absurdity; it was provided formerly but not in our day; the people read the newspapers and magazines which have become a power in the land; by this reading they keep up with the intelligence of the times, and the clergymen who succeed must have the means of doing the same.

